# Unsuccessful Application of a Ligature on the Femoral Artery for a Wound of the Anterior Tibial, Followed by Amputation

**Published:** 1850-01

**Authors:** W. S. Davis

**Affiliations:** Heytesbury


*Unsuccessful Application of a Ligature on the Femoral Artery for a Wound of the Anterior Tibial, followed by Amputation.* By W. S. Davis, Esq., M.R.C.S. &c., Heytesbury.—A case which occurred in my practice during the last summer bears so much on a point of surgery mooted in the lectures of that high authority, Mr. Guthrie, and commented on in your journal lately, by Dr. Massey, of Nottingham, that, though I am but a young *country practitioner*, I am tempted to publish it, begging you to consider it as taken from a kind of general note book for my own private use, and consequently deficient in detail.

Aug. 4th, 1848.—William P——, aged twenty-eight, robust and temperate, a farm-labourer, whilst engaged in reaping, allowed his very sharp hook to dangle carelessly between his thumb and finger; whilst hitting at a few stray wheat straws the point was driven deeply into the anterior part of the leg.

I was summoned to him, and being from home did not see him (two miles from my house) till about two hours after the accident. I found him seated at his cottage, with the wounded leg in a chair, before *a good fire*, and very faint from loss of blood. I learnt that tobacco had been applied to the wound, which was tightly bound with handkerchiefs in the field, by a bystander, which effectually stayed the hæmorrhage. All that I could ascertain in addition was, that "he had lost some pints of blood, which came from him as from a stuck pig."

I presumed that an artery had been wounded, however, from his depressed state. I merely saw him removed to bed, did not interfere with the bandages, and applied a tourniquet loosely over the upper third of femoral artery, giving directions as to tightening, should bleeding return, ordering him to be kept cool, &c. I visited him again some four hours after, found him rallied; no return of hæmorrhage.

August. 5th.—No oozing of blood. To be kept very quiet, and carefully watched.

6th & 7th.—No uneasiness about the wound, which was *not* interfered with. No oozing of blood.

8th.—Complained much of stiffness of bandages, pain, &c. from tightness. Removed bandages, dressed wound, &c.

9th.—Wound looking pretty well; no return of hæmorrhage. Ordered him to be kept low, and in the same position as before.

10th.—I was summoned in great haste at four A. M. Found the patient nearly sunk from loss of blood, which had flowed in a considerable stream. His wife had tightened the tourniquet, and after some flurry had succeeded in stopping it; it had escaped in sufficient quantities to have almost saturated the sheets.

Satisfied that an artery was wounded, and that the tourniquet commanded the flow of blood, I made up my mind to tie the femoral, and rode after a valued friend, an experienced surgeon of twenty years' extensive practice, who kindly came and assisted me in the operation, which was performed at six A. M., the patient being under the influence of chloroform. The limb was afterwards wrapped in flannel, &c. Saw him twice again on the same day, and found him comfortable. On the two succeeding days he went on well.

13th —Was summoned to my patient about six P. M., and found considerable arterial bleeding from the accidental wound. In consultation with my friend I applied tannin, Ruspini's styptic, bandage, &c.

14th.—No fresh hæmorrhage. Applications seemed to have effect.

15th.—Was summoned about nine P. M., and, in consultation with my friend, finding he was much reduced from an additional loss of blood, and darkness coming on, I re-plugged the wound, and left it till morning.

16th.—An experienced surgeon of many years' standing met us early in consultation, when we agreed to remove plugs and enlarge wound, endeavoring to find ends of bleeding vessel or vessels; and if we failed, to amputate the leg below the knee, as a last resource. There had been no escape of blood since the previous night, but on removing plugs, &c., hæmorrhage burst forth with increasing violence. I proceeded as we agreed, but found the difficulty consequent on state of parts; we could not command the flow of blood sufficiently to justify delay, and I performed the circular amputation as quickly as I could, the patient being under the influence of chloroform. A large quantity of blood was lost. I remained within a few yards of him during day and night, but *no hæmorrhage* followed this last operation. From this time everything proceeded satisfactorily; there was a slight exfoliation at the edge of the divided tibia, yet the stump healed kindly, and he is now earning a good living as a "pike-keeper."

*Remarks.*—I have had the opinion of two or three hospital surgeons, of very high standing, that the treatment pursued was proper. I give the words of one:—" I am not prepared to say that it could have been better managed. In nine cases out of ten your first operation of ligaturing the superficial femoral artery would have sufficed to control all hæmorrhage, but the tenth case (such I consider yours) has induced surgeons of authority to lay down the rule, that an artery when wounded, should be cut down upon, and both ends secured; of course there are exceptions to every general rule, and your case might fairly be considered one."

I have not the presumption to give an opinion on the practice with such high authorities, but I can only say, from the disappointment the first operation occasioned me, that I should, in a similar case, follow the principle laid down by Mr. Guthrie. It is some consolation to me to know that an hospital surgeon now in practice, and whom three-fourths of the profession would, I believe, place at the very top of the tree, failed to find and secure bleeding vessels in the same situation!

Some may think I might have tied the popliteal, others, that the femoral should have been secured *above the profunda.* The reasons for not choosing the former operation are well explained by Dr. Massey in his paper. In the latter case, I acknowledge, my patient might have escaped with his limb; but I would ask any surgeon—Had I not a right to expect I had secured him by tying the superficial femoral?

With regard to the wounded vessel or vessels, I am induced to believe that more than one was injured, as the principal vessels of the leg are so close to each other, and which rendered my *second* operation far more difficult. Many, perhaps, will say that it could have been easily ascertained in the amputated limb. I would, with all deference, ask those who know the inconvenience of surgical operations, dissections, &c., in a confined country cottage,—where you have not only innumerable disadvantages to put up with, but the prejudices of relatives against your removing the limb from their sight, or making one more incision than perfectly necessary, though a dead member, believing in their ignorance, curiosity to be your motive, if it is to be wondered at that I did not ascertain it? I *did* make a quick dissection of the limb in an adjoining room, but, from the hurried and rough way in which I was obliged to proceed, I will not advisedly assert that I avoided wounding another branch, besides anterior tibia, during the process!

Should any gentleman favour me with a notice of the case in your valuable journal, I shall be pleased to supply any additional particulars he may require to this imperfect statement!

In Dr. Massey's case the patient is described as suffering for many years from large ill-conditioned ulcers on his extremeties. My patient, a large framed, robust, and healthy fellow, had never suffered from *palpitation* or other ailments. Could the condition of the two have caused so marked a difference in freedom of anastomosis!—*Lancet.*

